# An R3-MYB repressor, BnCPC forms a feedback regulation with MBW complex to modulate anthocyanin biosynthesis in *Brassica napus*

**DOI:** 10.1186/s13068-022-02227-6

**Published:** 2022-11-29

**Authors:** Tao Xie, Xiongyun Zan, Xin Chen, Haotian Zhu, Hao Rong, Youping Wang, Jinjin Jiang

**Affiliations:** 1 Jiangsu Provincial Key Laboratory of Crop Genetics and Physiology, Yangzhou University, Yangzhou, 225009 China; 2 Joint International Research Laboratory of Agriculture and Agri-Product Safety, The Ministry of Education of China, Yangzhou, 225009 China; 3 School of Biological and Food Engineering, Suzhou University, Suzhou, 234000 China

**Keywords:** *Brassica napus*, Anthocyanin, BnCPC, MBW complex, Repressor, *BnDFR*

## Abstract

**Background:**

Anthocyanins are metabolites of phenylpropanoid pathway, and involves in diverse processes of plant development and adaptation, which are regulated by the MYB-bHLH-WD40 (MBW) protein complexes. Many R2R3-MYB activators have been well characterized, but the MYB repressors in anthocyanin biosynthesis were recognized recently, which are also important in modulating phenylpropanoid metabolism in plants. The regulatory mechanism of anthocyanin biosynthesis in oil crop *Brassica napus* remains to be revealed.

**Results:**

In this study, we identified an anthocyanin repressor BnCPC in *B. napus*. *BnCPC* encoded a typical R3-MYB protein containing a conserved [D/E]Lx2[R/K]x3Lx6Lx3R motif for interaction with bHLH proteins. Overexpression of *BnCPC* in *B. napus* inhibited anthocyanin accumulation, especially under anthocyanin inducible conditions. Protein–protein interaction and dual-luciferase assays confirmed that BnCPC could compete with BnPAP1 to interact with bHLHs (BnTT8 and BnEGL3), and repress the expression of anthocyanin biosynthetic genes (e.g., *BnDFR*) that activated by MBW complexes. Moreover, we found BnCPC inhibited the MBW complex-induced *BnCPC* activity.

**Conclusions:**

Overall, this research demonstrated that BnCPC repressed anthocyanin biosynthesis by affecting the formation of MBW complex, and formed a feedback loop to regulate anthocyanin accumulation in *B. napus*.

**Supplementary Information:**

The online version contains supplementary material available at 10.1186/s13068-022-02227-6.

## Introduction

The colorful plant kingdom contains a wide variety of natural pigments that impart different colors to tissues and organs, such as leaf, stem, flower, fruit, and seed [1, 2]. Anthocyanins, betalains, and carotenoids are common natural pigments that play key roles in plant development and reproduction [3, 4]. Anthocyanins are a kind of water-soluble natural pigments in plants that are responsible for a wide range of colors ranging from orange/red to violet/blue [3, 5]. The anthocyanin accumulation contributes to plant pollination and seed dispersal, confers plant resistance to pest diseases, UV radiation, pathogen infection and herbivores, and can significantly improve plant tolerance to abiotic stresses [6–10]. Furthermore, anthocyanins are proved with potential health advantages [11, 12]. Due to its antioxidant properties, anthocyanins play essential roles in improving human immunity, body weight regulation, anti-aging, anti-cancer, and other health fields [13–15].

Anthocyanins are synthesized via the flavonoid branch of phenylpropanoid pathway, and the genes related to anthocyanin biosynthesis are categorized into early biosynthesis genes (EBGs) and late biosynthesis genes (LBGs) [16, 17]. The EBGs include *chalcone synthase* (*CHS*), *chalcone isomerase* (*CHI*), *flavanone 3-hydroxylase* (*F3H*), and *flavanone 3′-hydroxylase* (*F3′H*), which are involved in precursor biosynthesis for flavonoids. The LBGs such as *dihydroflavonol 4-reductase* (*DFR*), *leucoanthocyanidin dioxygenase* (*LDOX*), and *UDP-glucose: flavonoid 3-O-glucosyltransferase* (*UF3GT*) are involved in anthocyanin biosynthesis [18]. Since anthocyanins play important roles in absorbing photosynthetic light energy and in plant response to biotic and abiotic stresses, molecular regulation of anthocyanin biosynthesis is valuable to achieve a balance between anthocyanin and proanthocyanidins (PAs), another main product of the flavonoid biosynthetic pathway. Furthermore, strict regulation of anthocyanin content is necessary to balance photoprotection and light absorption in plants [10, 19]. In *Arabidopsis*, the EBGs are modulated by subgroup 7 R2R3-MYB transcription factors (*MYB11*, *MYB12*, and *MYB111*) [16, 20, 21], and the activation of LBGs requires MYB-bHLH-WD40 (MBW) protein complexes [20, 22, 23]. The R2R3-MYBs are key regulators in the spatial and temporal patterns of anthocyanin localization and deposition in plants [20]. Anthocyanin-related R2R3-MYB activators have been widely identified in various plants, such as *AtMYB75* (*PAP1*), *AtMYB90* (*PAP2*), *AtMYB113*, and *AtMYB114* in *Arabidopsis* [22, 24–26], *VvMYBA1*, *VvMYBA2*, *VvMYBA5*, *VvMYBA6*, and *VvMYBA7* in grape [27, 28], *MdMYB1*, *MdMYB3*, *MdMYB10*, and *MdMYB110a* in apple [29–32], *PtrMYB57*, *PdMYB118*, and *PtrMYB119* in poplar [33–35]. These R2R3-MYBs mainly regulate anthocyanin biosynthesis through modulating the gene expression in flavonoid biosynthetic pathway. For instance, AtMYB75/90/113/114 formed complexes with bHLHs (GL3, EGL3, and TT8) and WD40 (TTG1) to regulate *LDOX* and *DFR* expression [36–38].

In addition to MYB activators, MYB repressors are also involved in regulating anthocyanin biosynthesis, including members of R2R3-MYB and R3-MYB [39]. Currently, anthocyanin-related MYB repressors have been identified in *Arabidopsis*, grape, and poplar [40]. The repressive activity of R2R3-MYBs was dependent on the repressive motif in the C-terminal [19]. For example, AtMYB4 in *Arabidopsis*, FaMYB1 in strawberry, PhMYB27 in petunia, and MaMYB4 in banana contained an ethylene-responsive element-associated amphiphilic repression (EAR) motif in the C-terminal domain, which was considered as a major domain repressing the transcription of anthocyanin structural genes [41–43]. TLLLFR motif, another conserved C-terminal domain was also existed in some R2R3-MYB (e.g., PtrMYB182 in *Populus*) and R3-MYB (e.g., AtMYBL2 in *A. thaliana*) repressors [44, 45]. AtMYBL2 negatively regulated anthocyanin biosynthesis through repressing *DFR* and *TT8* expression [40, 44]. Other R3-MYB proteins, such as PhMYBx in petunia and AtCPC in *Arabidopsis* only contained a bHLH-binding motif in the R3 domain, and no repressive region has been identified with binding ability to the promoters of anthocyanin biosynthesis genes [10, 46]. In *Arabidopsis* and lily (*Lilium* spp.), the R3-MYB repressors affected the *DFR* promoter activity by binding bHLH protein as a competitive inhibitor, and affected the formation of MBW complex and inhibited anthocyanin accumulation [46, 47]. For instance, AtCPC repressed anthocyanin biosynthesis by competing with AtMYB75/90 to bind AtGL3/AtEGL3 and affecting MBW formation [46]. In grape hyacinth, MaMYBx regulated anthocyanin biosynthesis through binding to MabHLH1 and disrupting the MaMybA/MaAN2-MabHLH1 complex, and was able to repress flower pigmentation in tobacco [48]. Nevertheless, little is known about how MBW activators and MYB repressors cooperated in the anthocyanin regulatory network to precisely control flavonoid content in plants.

Rapeseed (*Brassica napus* L., 2*n* = 38) is a natural allotetraploid with great economic values and is widely cultivated as an oil crop in the world [49]. Due to the genome complexity compared with *Arabidopsis*, the regulation mechanism of anthocyanin biosynthesis in *B. napus* is more complicate and it has not been fully elucidated yet. Although many differentially expressed genes (DEGs) have been reported among rapeseed materials with different seed, leaf, or flower colors [50–52]. Hitherto, the functionally reported genes affecting anthocyanin biosynthesis in *B. napus* are structural genes (e.g., *DFR, FLS,* and *LDOX*) [52, 53] and transcription regulatory factors (PAP2.A7, BnGL3-1, and WRKY41-1) [54–57]. The MYB repressors involved in anthocyanin biosynthesis of *B. napus* have not been reported. Herein, we characterized BnCPC as an R3-MYB repressor that negatively regulated anthocyanin accumulation in *B. napus*. We found that BnCPC affected the formation of MBW complexes by interacting with bHLH proteins (BnTT8 or BnEGL3), thereby inhibiting the expression of anthocyanin biosynthetic genes. Furthermore, BnCPC was able to inhibit the MBW complex-induced *BnCPC* activity, to form a feedback regulation with MBW complexes in regulating anthocyanin biosynthesis.

## Results

### BnCPC encodes a CPC-type R3-MYB protein

**Fig. 1:**
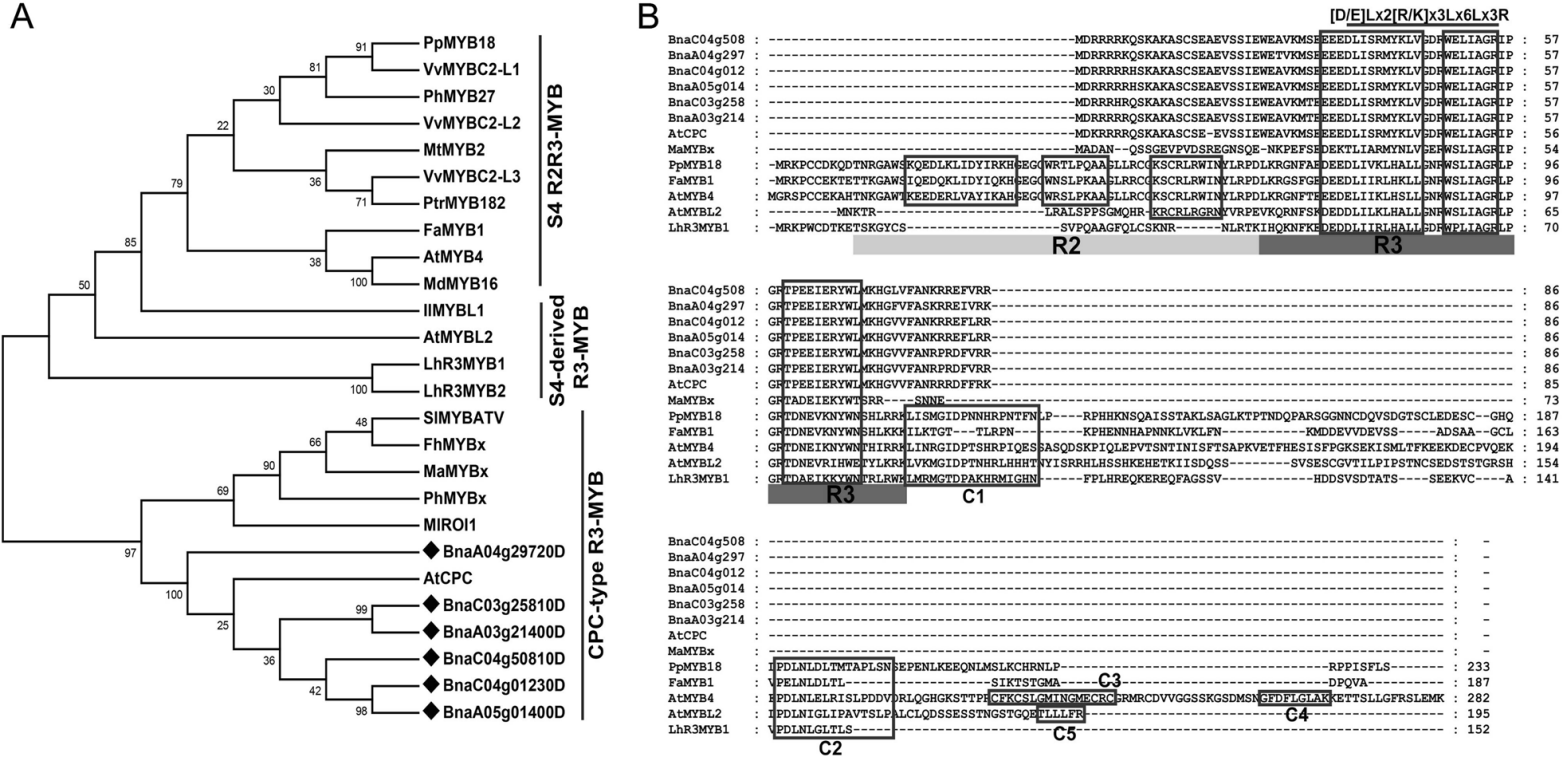
Characterization of BnCPC proteins. **A** Phylogenetic relationship of BnCPC and other known MYB repressors in plants. BnCPC homologues were highlighted with the black rhombi. Alignment of full amino acid sequences of MYB repressors was done by MUSCLE software, and phylogenetic tree was constructed using the neighbor-joining method by MEGA software 7.0. Numbers at the nodes indicated the reliability percentage of bootstrap values based on 1000 replications. **B** Alignment and structural domains of BnCPC and MYB repressors. Protein sequences were aligned using ClustalX. The R2 and R3 domains were indicated with grayish white and gray boxes, respectively. Three α-helices of both R2 and R3 domains were indicated in boxes. The conserved MYB–bHLH interaction motif ([D/E]Lx2[R/K]x3Lx6Lx3R) of R3 domain was underlined with a black bar. The conserved motifs in the C-terminus were highlighted with boxes and numbers. C1 domain, LIsrGIDPxT/SHRxI/L; C2 domain (the EAR repression motif), pdLNLD/ELxiG/S; C3 domain (zinc-finger motif), Cx2Cx9CxC; C4 domain, GY/FDFLGL motif; C5 domain, TLLLFR repressor motif. The following GenBank or *Arabidopsis* TAIR accession numbers were used, PpMYB18 (KT159234.1), VvMYBC2-L1 (JX050227), VvMYBC2-L2 (ACX50288), VvMYBC2-L3 (KM046932), FaMYB1 (AF401220), MtMYB2 (XM_003616340), PtrMYB182 (KP723392), AtMYB4 (AF062860), PhMYB27 (AHX24372), MdMYB16 (HM122617), AtMYBL2 (AEE35154), IlMYBL1 (ASR83103.1), LhR3MYB1 (BBG71951.1), LhR3MYB2 (BBG71953.1), AtCPC (AT2G46410.1), PhMYBx (AHX24371.1), MlROI1 (AGC66791.1), SlMYBATV (NP_001352307), FhMYBx (MT210095), MaMYBx (QJH86892.1)

Six *BnCPC* homologues were identified in the rapeseed genome, which encoded proteins with 86 amino acids in length and contained a conserved R3-MYB domain. A phylogenetic analysis of known MYB repressors suggested that BnCPCs belonged to the CPC-type R3-MYB family, and were most conserved to AtCPC (Fig. 1A), which was a negative regulator of anthocyanin accumulation in *Arabidopsis* [46]. Multiple sequence alignment revealed that BnCPC contained an N-terminal R3-DNA binding domain with a conserved motif, [D/E]Lx2[R/K]x3Lx6Lx3R, for interaction with bHLH proteins (Fig. 1B). Other repressive domains such as EAR or TLLLFR motif were not found in BnCPCs, agreeing with the characteristics of other reported R3-MYB anthocyanin inhibitors (e.g., AtCPC and MaMYBx) [46, 48]. Based on the transcriptome data of *B. napus* from the BnTIR database, we found the *BnCPC* homologues were highly expressed in root and developing seeds, and BnaC04g50810D was highly expressed than other homologues at the transcriptional level (Additional file 1: Fig. S1A), which was used for functional analysis of *BnCPC* in this research. Transient expression assay of *35S::eGFP-BnCPC* in tobacco leaves showed that BnCPC was a nuclei-localized transcription factor (Additional file 1: Fig. S1B). These findings indicated that BnCPC encoded a putative anthocyanin-related R3-MYB repressor in rapeseed.

### Analysis of *BnCPC* expression in anthocyanin inducible conditions

**Fig. 2:**
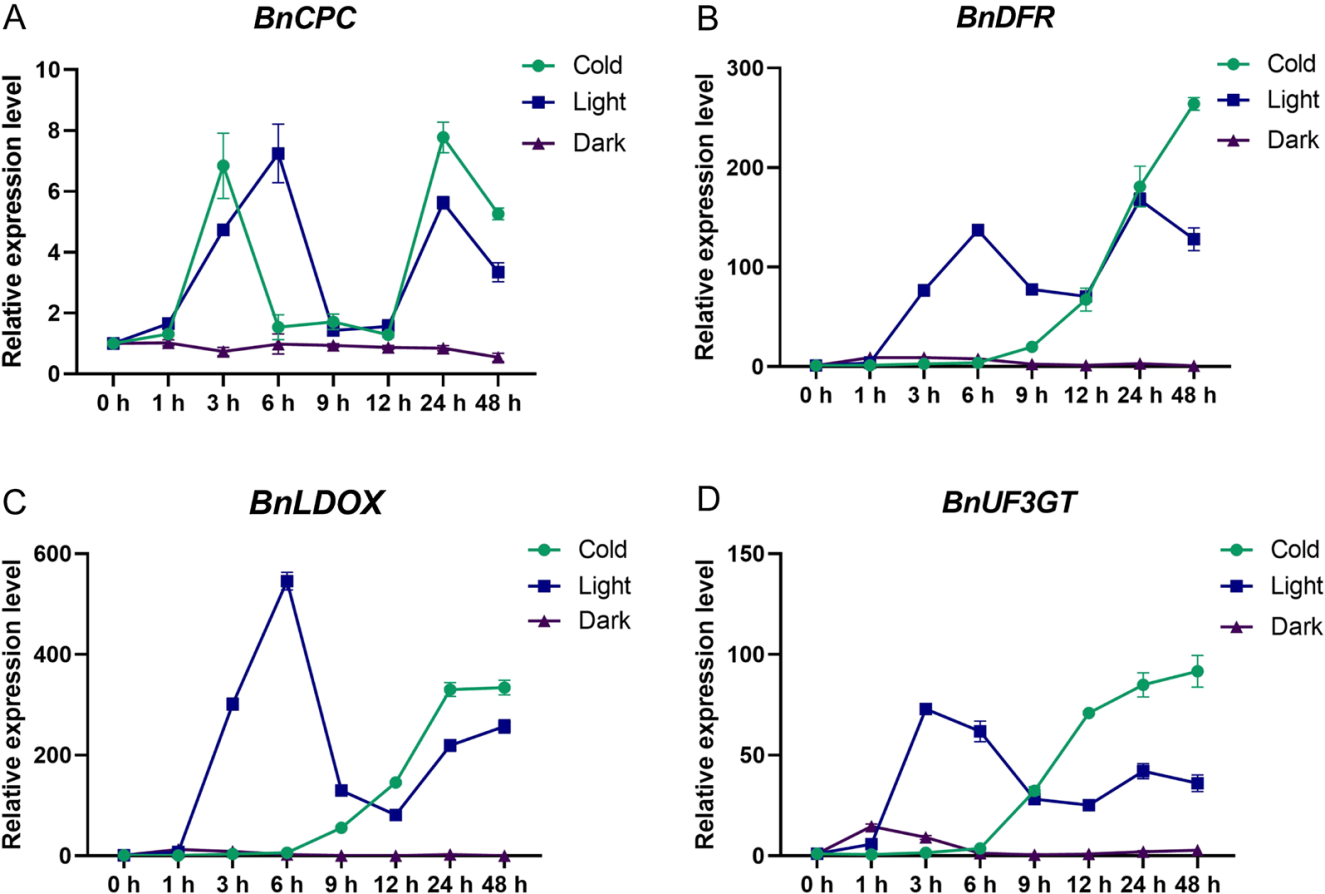
The expression pattern of **A** *BnCPC* and **B–D** anthocyanin biosynthetic genes (*BnDFR*, *BnLDOX*, and *BnUF3GT*) under anthocyanin inducible (cold and light) and non-inducible (dark) growth conditions. Expression levels were standardized to *B. napus Actin-7* (NC_027775.2), and the expression levels before treatment were set at 1. Values represented the mean ± SD (*n* = 3)

To understand the putative function of *BnCPC* in anthocyanin biosynthesis, we analyzed *BnCPC* expression pattern under anthocyanin inducible (cold and light) and non-inducible (dark) growth conditions, and found *BnCPC* was significantly induced after 3 h, 6 h and 24 h of cold treatment or light treatment, while no significant change was identified under dark treatment (Fig. 2A). Furthermore, we found the expression pattern of three anthocyanin biosynthetic genes, *BnDFR*, *BnLDOX,* and *BnUF3GT*, were similar to *BnCPC*, which were significantly up-regulated after 3 h, 6 h, and 24 h of light treatment, while these biosynthetic genes were continuously induced by cold treatment (Fig. 2B–D). These results indicated that *BnCPC* was up-regulated as the anthocyanin biosynthetic genes under the anthocyanin inducible conditions, suggesting that BnCPC might be correlated with anthocyanin biosynthesis in rapeseed.

### *BnCPC* overexpression reduces anthocyanin production in rapeseed

**Fig. 3:**
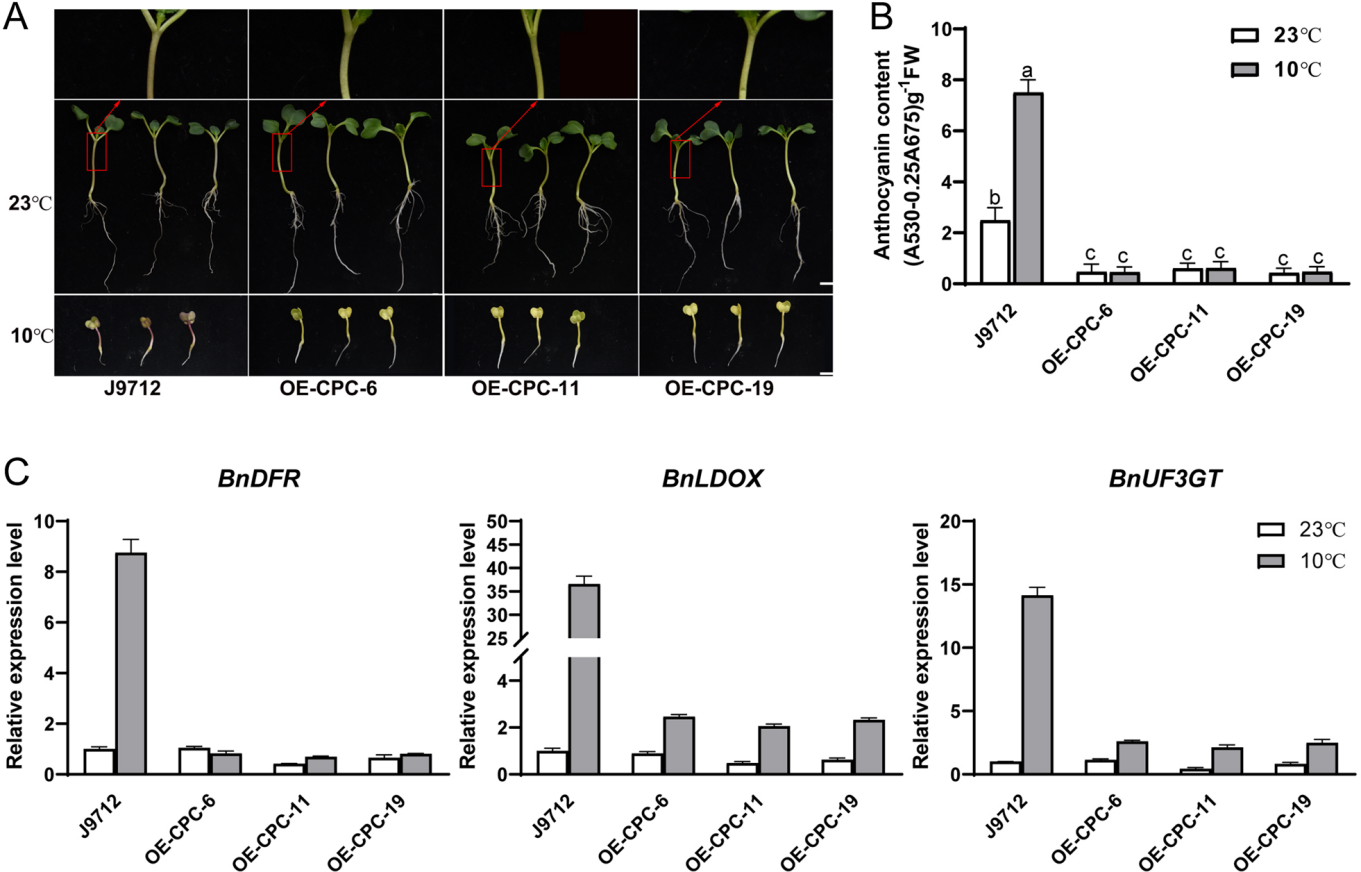
BnCPC repressed anthocyanin accumulation in *B. napus*. **A** Phenotype of J9712 and *BnCPC* overexpression (OE-CPC) lines under low temperature (10 °C) and normal temperature (23 °C). Scale bar represented 1 cm. **B** Anthocyanin content in extracts from seedlings in **A**. (A_530_-0.25 × A_657_)/gram fresh weight was considered as the relative anthocyanin content. Three biological replicates were performed, and 10 plants were pooled as one replicate. FW, fresh weight. Values represented the mean ± SD (*n* = 3). Different letters represented statistically significant differences (two-way ANOVA, *p* < 0.05). **C** The expression level of *BnDFR*, *BnLDOX*, and *BnUF3GT* in rapeseed seedlings from **A**. Expression levels were normalized to *B. napus Actin-7* (NC_027775.2), and the expression level of J9712 under normal temperature was set at 1. Values represented the mean ± SD (*n* = 3)

To investigate whether *BnCPC* is involved in regulating anthocyanin biosynthesis in *B. napus*, the cDNA of *BnCPC* (BnaC04g50810D) was overexpressed in rapeseed line J9712 under the control of CaMV *35S* promoter, and three independent overexpressed lines (OE-CPC-6, OE-CPC-11, and OE-CPC-19) were used for phenotypic analysis (Additional file 1: Fig. S2). Under normal growth conditions (23 °C, 16 h light/8 h dark), we found the anthocyanin accumulation in hypocotyls of 7-day-old OE-CPC seedlings was less than that in J9712. And the cold-induced anthocyanin accumulation in J9712 was not observed in *BnCPC* overexpression lines (Fig. 3A, B). On the basis of RNA-seq analysis, we found all the LBGs (e.g., *DFR*, *LDOX*, *UF3GT*, and *GSTF12*) in flavonoid biosynthetic pathway were downregulated in *BnCPC* overexpression lines compared to J9712 under cold treatment (Additional file 1: Fig. S3, Additional file 2: Table S1). Besides, the *F3′H* and the regulatory genes *BnTT8* and *BnPAP1* (*BnaCnn28030D*) were also significantly downregulated in OE-CPC lines. In addition, qPCR analysis also confirmed that *BnDFR*, *BnLDOX,* and *BnUF3GT* expression were inhibited in OE-CPC lines compared with J9712 when grown under 23 °C and 10 °C (Fig. 3C). These DEGs might be responsible for the reduced anthocyanin accumulation in *BnCPC* overexpression lines.

Furthermore, we analyzed the function of *BnCPC* in other growth conditions that induce anthocyanin biosynthesis, such as nitrogen deficiency (LN), sucrose, and jasmonic acid (JA) treatment. Under all tested stress and hormone treatments, the anthocyanin was more accumulated in the hypocotyls of J9712 than that grown in 1/2 MS or solid Hoagland medium with high nitrogen (HN), but it was barely accumulated in the OE-CPC lines grown under stress conditions (Additional file 1: Fig. S4A, B). The expression of anthocyanin biosynthesis related genes was repressed in *BnCPC*-overexpressed lines than J9712 when grown under nitrogen deficiency, sucrose and JA treatment. Meanwhile, we found *BnDFR* was much more repressed than other biosynthetic genes (Additional file 1: Fig. S4C). These results proved that BnCPC was a repressor of anthocyanin biosynthesis in rapeseed seedlings.

### BnCPC represses the MBW complex-induced *BnDFR* activity

**Fig. 4:**
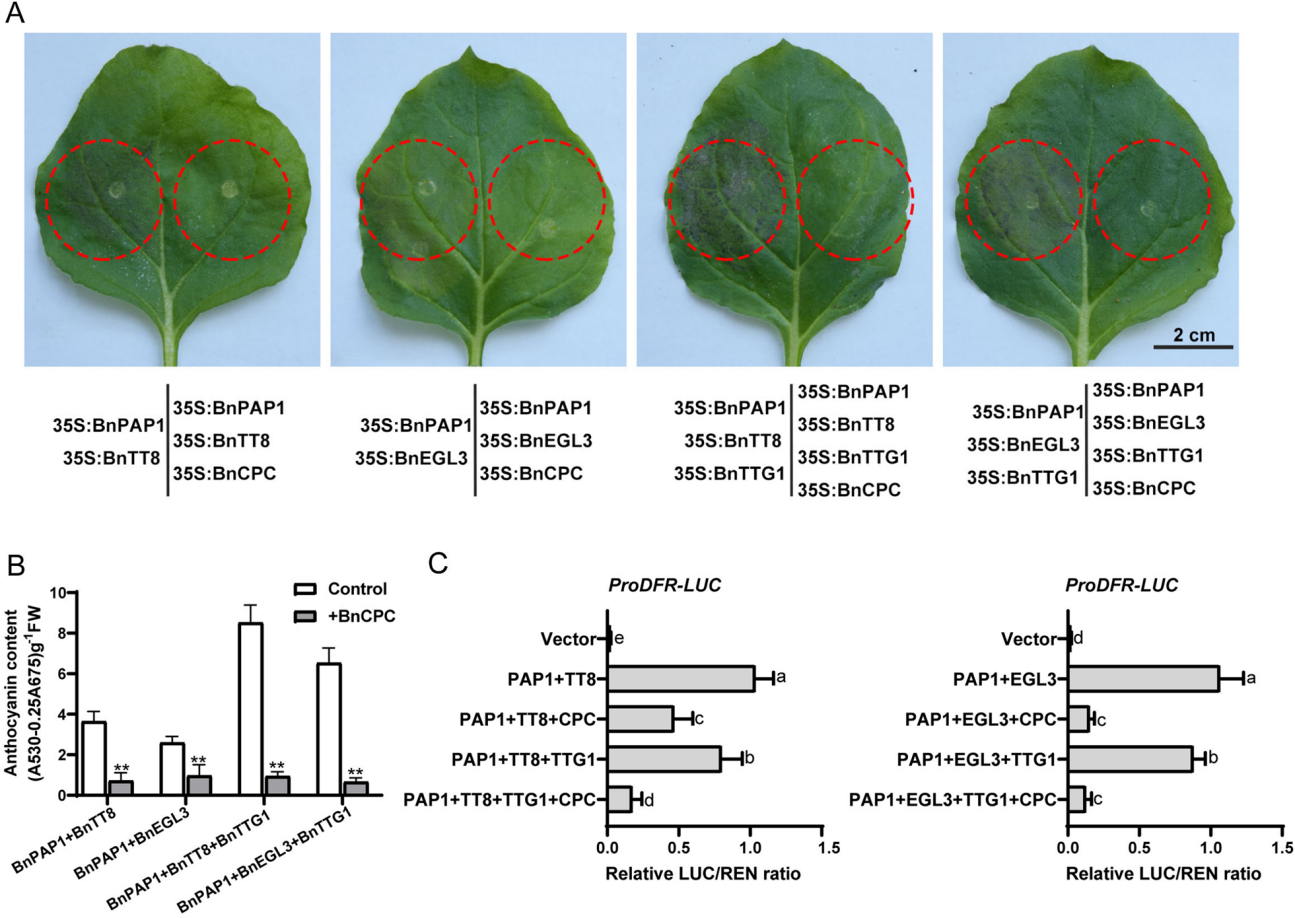
BnCPC repressed the *BnDFR* promoter activity by interfering with the formation of MBW complexes. **A** BnCPC inhibits anthocyanin accumulation mediated by MBW complexes in tobacco leaves. Tobacco leaves were infiltrated with BnPAP1 + BnTT8 ± BnCPC, BnPAP1 + BnEGL3 ± BnCPC, BnPAP1 + BnTT8 + BnTTG1 ± BnCPC, BnPAP1 + BnEGL3 + BnTTG1 ± BnCPC and cultured for 8 days. **B** Relative anthocyanin content in extracts from leaves in **A**. Independent-samples *t*-test was used for statistical analysis in comparison with the controls (**p ≤ 0.01). **C** BnCPC inhibited the MBW complex-induced *BnDFR* promoter activity. Values represent the mean ± SD (*n* = 6). Different letters represented statistically significant differences (one-way ANOVA, *p* < 0.05)

We used transient expression assay in tobacco to analyze the putative molecular mechanism of BnCPC in repressing anthocyanin biosynthesis, and found co-infiltration of *35S:BnPAP1* with *35S:BnTT8* or *35S:BnEGL3* induced purple pigmentation in tobacco leaves, but no pigmentation was observed when *35S:BnPAP1* + *35S:BnTT8* or *35S:BnPAP1* + *35S:BnEGL3* were co-infiltrated with *35S:BnCPC*. Besides, co-transformation of *35S:BnTTG1* enhanced the pigmentation induced by *35S:BnPAP1* + *35S:BnTT8*/*35S:BnEGL3*, which were also inhibited by *35S:BnCPC* (Fig. 4A). Quantitative analysis also confirmed the function of BnPAP1-BnTT8/BnEGL3-BnTTG1 in inducing anthocyanin biosynthesis, and the role of BnCPC as an anthocyanin repressor (Fig. 4B). This agreed with the previous studies that CPC-type R3-MYBs play as repressors in anthocyanin biosynthesis by preventing the formation of MBW complexes [19, 40].

We used dual-luciferase reporter assay in tobacco to test the effect of BnCPC on anthocyanin biosynthetic genes, and found that co-transformation of BnPAP1 + BnTT8 or BnPAP1 + BnTT8 + BnTTG1 significantly enhanced the activity of *BnDFR* promoter. But the *BnDFR* promoter activity was significantly repressed when BnCPC was co-introduced with BnPAP1 + BnTT8 and BnPAP1 + BnTT8 + BnTTG1. Besides, BnCPC also repressed the *BnDFR* activity induced by BnPAP1 + BnEGL3 and BnPAP1 + BnEGL3 + BnTTG1 (Fig. 4C). Thus, we may speculate that BnCPC inhibits the activity of anthocyanin biosynthetic genes by influencing MBW complexes.

### BnCPC interacts with bHLH proteins of MBW complexes

**Fig. 5:**
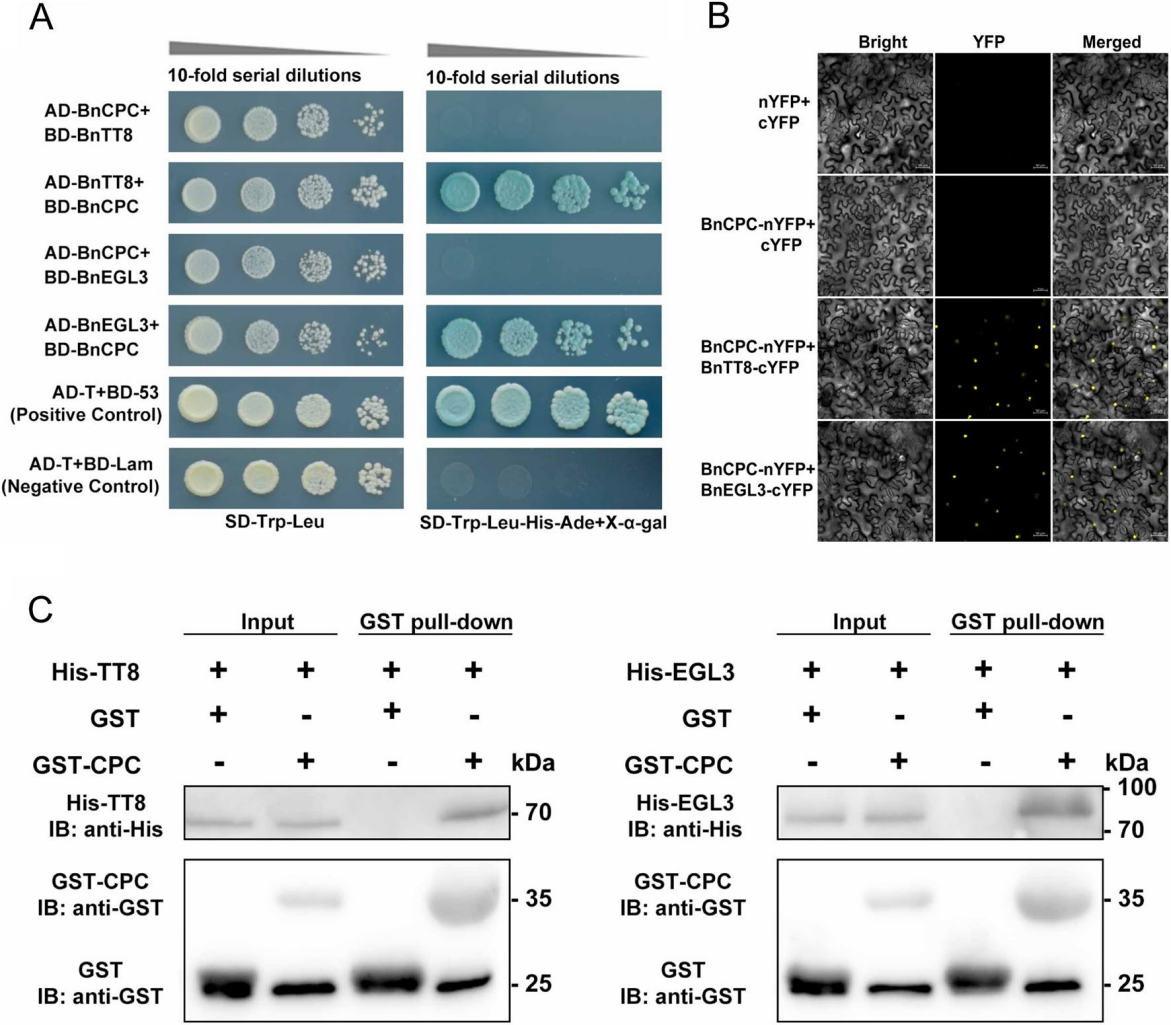
BnCPC interacts with BnTT8 and BnEGL3. **A** Interaction of BnCPC with BnTT8/BnEGL3 by Y2H assay. **B** Interaction of BnCPC with BnTT8/BnEGL3 by BiFC assay. **C** Interaction of BnCPC with BnTT8/BnEGL3 by pull-down assay

As mentioned above, BnCPC contained a conserved motif for bHLH interaction, and it also affected the *BnDFR* activity by influencing MBW complexes. Here we adopted yeast two-hybrid (Y2H) assay to analyze the interactions between BnCPC and BnTT8/BnEGL3, aiming to illustrate how BnCPC affects the function of anthocyanin-related MBW complexes. The results showed that BnCPC interacted with BnTT8 and BnEGL3 in yeast cells (Fig. 5A). Bimolecular fluorescence complementation (BiFC) assay confirmed the interaction between BnCPC and BnTT8/BnEGL3 (Fig. 5B). GST pull-down assay was also used to validate the interaction between BnCPC and BnTT8/BnEGL3 (Fig. 5C). These results revealed that BnCPC physically interacted with BnEGL3 and BnTT8 both in vitro and in vivo.

### BnCPC competes with BnPAP1 to affect MBW formation

**Fig. 6:**
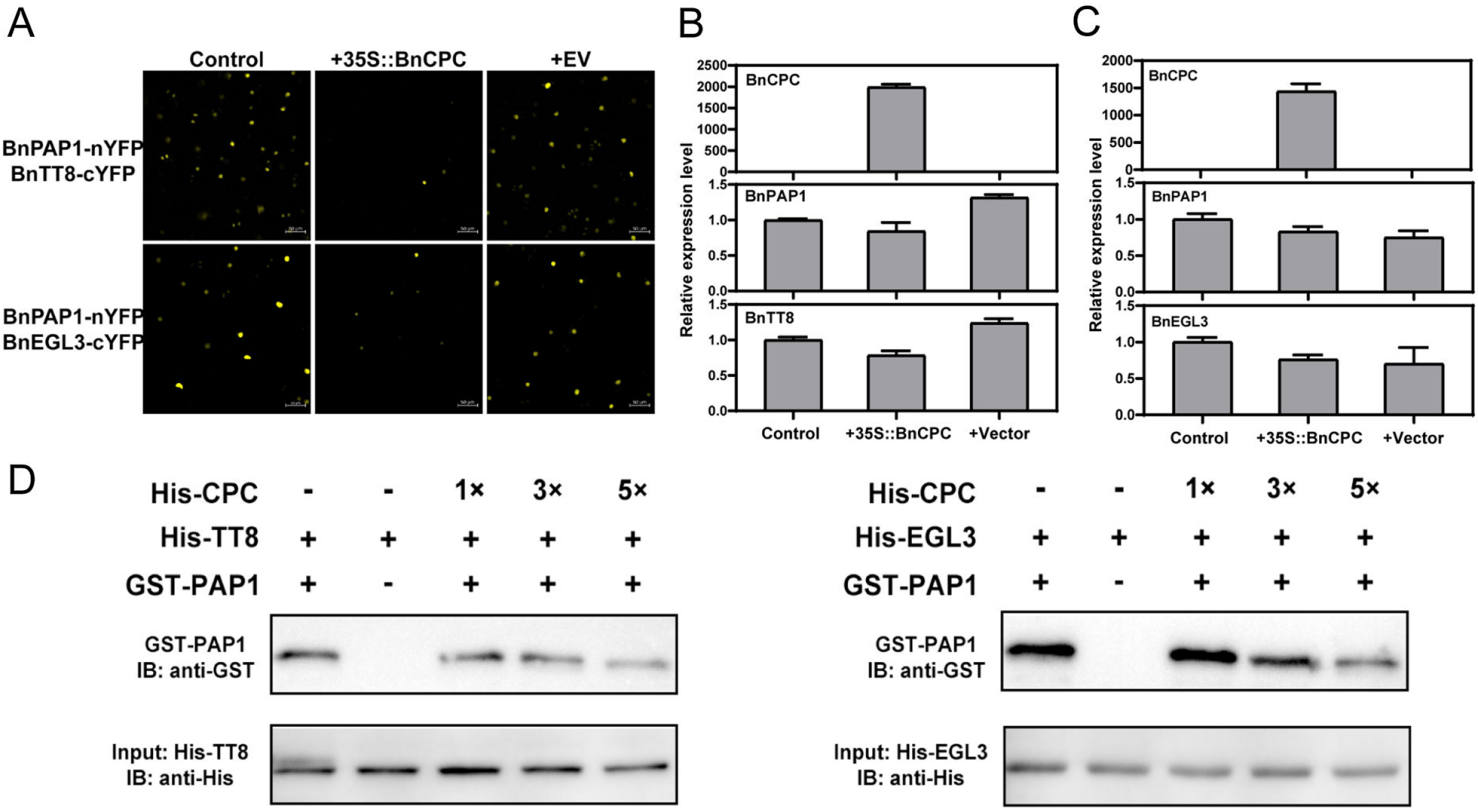
BnCPC competes with BnPAP1 to affect MBW formation. **A** BiFC analysis showed that BnCPC affected the interaction of BnPAP1 with BnTT8/BnEGL3. YFP fluorescence was observed 48 h after co-expression. Scale bar represented 100 μm. **B** and **C** qPCR analysis of *BnCPC*, *BnTT8*, *BnEGL3*, and *BnPAP1* expression in **A**. Expression level was standardized to tobacco *Actin* (JQ256516.1). Values represented the mean ± SD (*n* = 3). **D** Competitive binding assays of BnCPC and BnPAP1 for BnTT8/BnEGL3

We conducted competitive binding assays to analyze whether BnCPC affects the MBW complex by competing with the MYB component (e.g., BnPAP1). Strong yellow fluorescent protein (YFP) signals were detected in nuclei when BnPAP1-nYFP and BnTT8-cYFP were transiently co-expressed in tobacco leaves. But it was impaired after *35S::BnCPC* was co-transformed with BnPAP1-nYFP and BnTT8-cYFP. Similar results were observed when *35S::BnCPC* was co-expressed with BnPAP1-nYFP and BnEGL3-cYFP (Fig. 6A). The expression level of *BnPAP1*, *BnTT8*, and *BnEGL3* were not significantly changed in the infiltrated tobacco leaves, even when *35S::BnCPC* was co-expressed (Fig. 6B, C). This confirmed that the binding ability between BnPAP1 and BnTT8 or BnEGL3 was affected by BnCPC, not by the expressional changes of BnPAP1/BnTT8/BnEGL3. We further tested whether BnCPC could interfere with the interaction between BnPAP1 and BnTT8/BnEGL3 with pull-down assay. Competitive binding experiments proved that the binding ability between BnPAP1 and BnTT8/BnEGL3 were impaired with the increase of BnCPC content (Fig. 6D). Taken together, these results demonstrated that BnCPC was capable to compete with BnPAP1 to bind BnTT8/BnEGL3, thus affected the formation of MBW complexes.

### BnCPC was induced by MBW complex

**Fig. 7:**
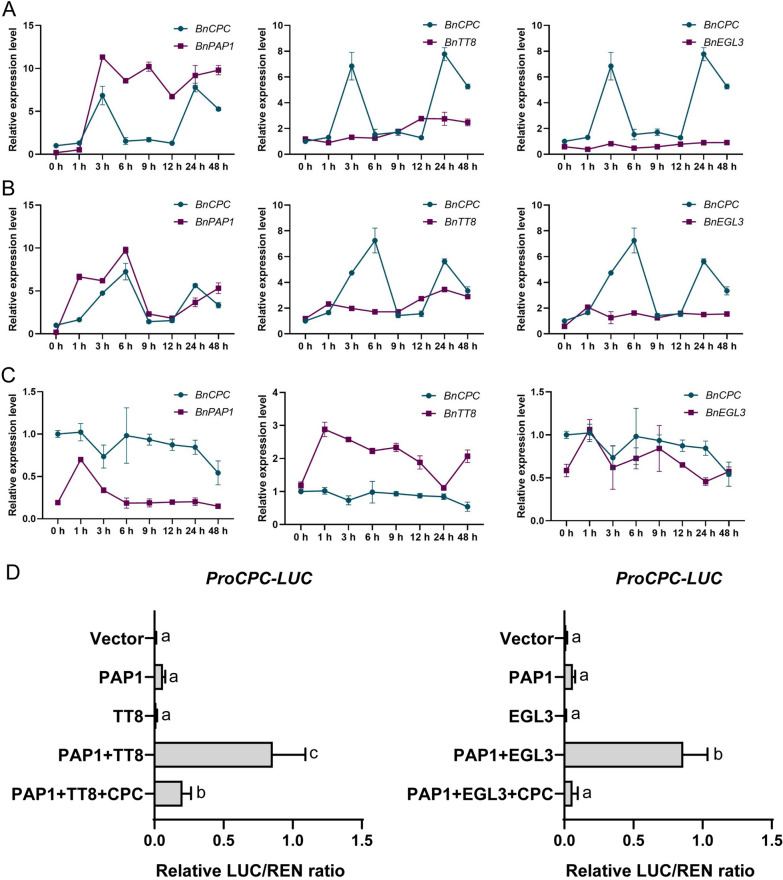
Regulatory relationships between *BnCPC* and the anthocyanin activators. **A–C** The expression pattern of *BnCPC*, *BnPAP1*, *BnTT8*, and *BnEGL3* under anthocyanin inducible (cold and light) and non-inducible (dark) growth conditions. Expression levels were standardized to *B. napus Actin-7* (NC_027775.2), and the expression levels of *BnCPC* before treatment were set at 1. Values represented the mean ± SD (*n* = 3). **D** Dual-luciferase assays of BnPAP1, BnTT8, BnEGL3, BnPAP1 + BnTT8/BnEGL3, and BnCPC + BnPAP1 + BnTT8/BnEGL3 effects on the activity of *BnCPC* promoter. Values represent the mean ± SD (*n* = 6). Different letters represented statistically significant differences (one-way ANOVA, *p* < 0.05)

To validate whether the components of MBW could regulate *BnCPC* expression to modulate appropriate anthocyanin biosynthesis, we analyzed the gene expression pattern under anthocyanin inducible (cold and light) and non-inducible (dark) conditions (Fig. 7A–C). qRT-PCR analysis revealed that both *BnCPC* and *BnPAP1* were significantly induced by cold and light treatment at 3 h cold and 6 h light treatments; while *BnCPC* and *BnPAP1* were not significantly induced under dark treatment. Unlike *BnPAP1*, *BnTT8* and *BnEGL3* expression were not induced by cold or light treatments. This suggested that the expression of *BnCPC* and *BnPAP1* were coordinately regulated by anthocyanin inducible growth conditions. Furthermore, we used dual-luciferase reporter assay to confirm the regulatory relationship among *BnCPC*, *BnPAP1*, *BnTT8*, and *BnEGL3*. We found that a single BnPAP1, BnTT8, or BnEGL3 was unable to enhance *BnCPC* promoter activity. While both BnPAP1 + BnTT8 and BnPAP1 + BnEGL3 significantly induced the promoter activity of *BnCPC*, which were repressed when BnCPC was co-transformed (Fig. 7D). This indicated that *BnCPC* could be induced by BnPAP1 and BnTT8/BnEGL3, the major components of MBWs. This agreed with previous reports that anthocyanin activators could activate MYB repressors, resulting in negative feedback regulation in anthocyanin biosynthesis [48, 50, 58]. Thus, BnCPC regulates anthocyanin biosynthesis by competing with BnPAP1 to bind BnTT8/BnEGL3 and affects MBW formation, and BnCPC could form a feedback regulation in inhibiting the MBW complex-induced *BnCPC* activity.

## Discussion

Anthocyanins are important secondary metabolites that could be induced by various environmental stimuli, such as strong light, low temperature, high sucrose concentration, drought stress, and hormones [59, 60]. For better adaptation to the changing environment, plants have evolved a series of mechanisms to regulate the anthocyanin biosynthesis. In most plants, anthocyanin accumulation is regulated by the MBW protein complexes, and R2R3-MYBs are pivotal to determine the MBW functions and the spatio-temporal localization/deposition of anthocyanins [20, 61]. Due to the genome complexity and lack of anthocyanin mutants, the regulatory network of anthocyanin biosynthesis has not been fully elucidated in rapeseed. Many putative genes associated with anthocyanin biosynthesis have been screened through comparative analysis of rapeseeds with color variations in different tissues (e.g., seed coat, leaf, and petal), including *BnDFR*, *BnLDOX*, *BnUF3GT*, *BnTT8*, *BnTT19* and *BnPAP2* [54, 62, 63]. However, the repressors involved in anthocyanin biosynthesis have not been reported in *B. napus*. Here, we characterized a MYB repressor BnCPC, which negatively regulated anthocyanin accumulation by inhibiting the function of MBW protein complexes.

MYB protein plays important roles in plant growth, development, and stress responses [64, 65]. In the anthocyanin biosynthetic pathway of plants, R2R3-MYB proteins are well known as activators by forming MBW complexes with bHLHs and WD40 [61]. A few MYB repressors involved in anthocyanin biosynthesis have been identified, including AtMYBL2 and AtCPC [44, 46], MtMYB2 in *Medicago truncatula* [66], MaMYBx in grape hyacinth [48], PhMYBx and PhMYB27 in petunia [42], and PpMYB18 in peach [50]. These MYB repressors could be divided into two types, R2R3-MYB and R3-MYB. In this study, sequence alignment showed that BnCPCs contained an R3-MYB and a bHLH-binding domain with high similarity to the anthocyanin repressors reported in *A. thaliana* (e.g., AtCPC) and grape hyacinth (e.g., MaMYBx) (Fig. 1B). In addition, the phylogenetic analysis grouped BnCPC to the CPC-type R3-MYB subgroup, which included multiple anthocyanin negative regulatory proteins (Fig. 1A). To elaborate the molecular function of BnCPC in *B. napus*, we created the *BnCPC* overexpression lines and analyzed the anthocyanin accumulation in response to various external stimuli, including low temperature, sucrose, nitrogen deficiency, and JA treatments (Fig. 2; Additional file 1: Fig. S4). The results showed that *BnCPC* negatively regulated anthocyanin accumulation, especially under stress and hormone treatments. And the expression of anthocyanin biosynthetic genes was also repressed, indicating that *BnCPC* repressed anthocyanin biosynthesis through inhibiting related genes in the biosynthetic pathway.

As reported, MYB repressors affect anthocyanin accumulation mainly through active and passive inhibitions [19, 40]. The active suppressors usually contain repressive motifs in the C-terminal, which is crucial for the repressive activities. For instance, PhMYB27 in petunia and MdMYB16 in apple were identified as repressors in anthocyanin biosynthesis, and deletion of the C-terminal EAR motif led to loss of function as anthocyanin repressors [42, 67]. Another inhibition domain TLLLFR motif, was identified in the C-terminus of AtMYBL2 [44], which also existed in other MYB repressors (e.g., FhMYB27, VvMYBC2, and PtrMYB182) [45, 68, 69]. However, both EAR and TLLLFR repressive motifs were not identified in the BnCPC proteins (Fig. 1B), which indicated that BnCPCs were not active repressors. Passive MYB repressors usually regulate anthocyanin biosynthesis through intermolecular interactions with bHLHs of the MBW complexes [19]. To date, all the reported MYB repressors in anthocyanin accumulation contain a conserved R3 domain with a [D/E]Lx2[R/K]x3Lx6Lx3R motif that interacts with bHLHs [19, 39, 40]. Furthermore, MYB repressors compete with MYB activators for binding to bHLH, suggesting that the bHLH-binding motif is critical for the inhibitory function of MYB repressors. Previously, mutations in the bHLH-binding motif disrupted the interactions of PpMYB18 in peach and PtrMYB182 in poplar with the bHLHs [45, 50]. Similarly, the motifs required for binding bHLHs were also identified in BnCPCs (Fig. 1B), and the interaction between BnCPC and bHLHs (e.g., BnTT8 and BnEGL3) were further confirmed by in vitro and in vivo protein–protein interaction assays (Fig. 5). The negative regulation of anthocyanin biosynthesis via affecting the MBW formation is common in plants [19, 40]. Based on the competitive BiFC and pull-down assays, we verified that BnCPC suppressed the interaction of MYB activator (BnPAP1) and bHLHs (BnTT8 or BnEGL3) by binding to the bHLHs, and affected the formation of MBW complexes (Fig. 6). Transient expression in tobacco leaves showed that BnCPC inhibited anthocyanin accumulation that induced by MBW complexes (Fig. 4A), indicating that BnCPC repressed the expression of anthocyanin biosynthetic genes by disrupting the MBW complexes. This hypothesis was confirmed by the *BnDFR* promoter activity analysis, which showed that BnCPC inhibited the *BnDFR* activity induced by BnPAP1-BnTT8/BnEGL3-BnTTG1 (Fig. 4C). In chrysanthemum, CmMYB#7 inhibited the transcriptional activation of anthocyanin biosynthetic genes by impairing the binding ability between CmMYB6 and CmbHLH2, while mutation of the bHLH-binding site on CmMYB#7 affected its repressive function on anthocyanin biosynthetic genes that activated by CmMYB6–CmbHLH2 complex [70]. Interestingly, the passive repressive mechanism was also identified in other transcription factors, such as HD-ZIP protein HAT1, SBP family protein SPL9, and JAZ proteins that inhibited anthocyanin accumulation by interacting with MYB activators or bHLHs, thus affecting the formation of MBW complexes [36, 37, 71].

In *Arabidopsis*, the expression of MYB repressors were correlated with anthocyanin accumulation or anthocyanin biosynthetic genes [40]. In *Citrus*, the expression of anthocyanin repressor *CsMYB3* was correlated with an R2R3-MYB activator *CsRuby1*, and anthocyanin accumulation in different tissues of *Citrus* and relative species [58]. Similarly, *PpMYB18* was predominantly expressed in the ripening fruit of blood-fleshed peach, but not expressed in the yellow- and white-fleshed fruits. Besides, the expression of anthocyanin repressor *PpMYB18* was correlated with the MYB activator *PpMYB10.1*, which was able to activate *PpUFGT* and *PpDFR* promotor activities [50]. In the present study, *BnCPC* was up-regulated in anthocyanin inducible conditions, with similar expression pattern correlated with anthocyanin biosynthetic genes (*BnDFR*, *BnLDOX*, and *BnUF3GT*) and regulatory gene *BnPAP1* (Figs. 2, 7A–C), suggesting a putative feedback loop via BnCPC in the regulation of anthocyanin biosynthesis. Thus, we speculated that *BnCPC* could be induced by the components of anthocyanin-related MBW complex. And it was further confirmed that BnPAP1 together with BnTT8/BnEGL3 could significantly activate the promoter of *BnCPC*, but it was inhibited in combination with BnCPC. This feedback regulation loop should be important in the appropriate regulation of anthocyanin biosynthesis in rapeseed. It is common that MYBs paly dual roles and could compete with each other to regulate flavonoid biosynthesis in plants. In *Arabidopsis*, MYB4 interacted with TT8, repressed the *MYB75/90* and *TT2* expression of MBW complexes, thus affected the MBW activity in regulating flavonoid biosynthesis [72].

**Fig. 8:**
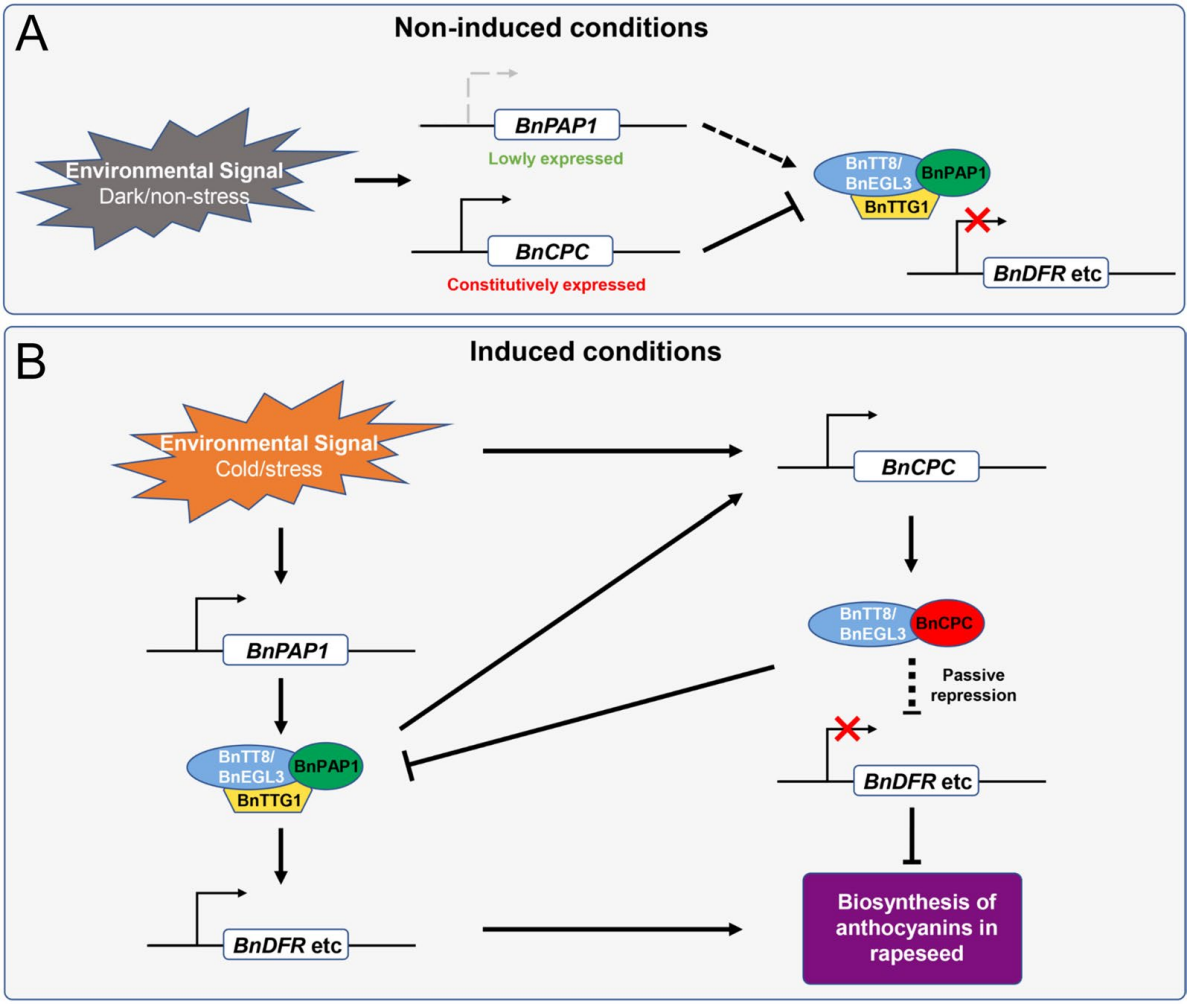
Proposed working model of BnCPC regulating anthocyanin biosynthesis in rapeseed. **A** Under non-inducible conditions (e.g., dark or non-stress conditions), *BnPAP1* was lowly expressed and *BnCPC* was continuously induced to repress the expression of anthocyanin biosynthetic genes (e.g., *BnDFR*) by inhibiting MBW formation, thereby repressed anthocyanin accumulation. **B** Under inducible conditions (e.g., cold and other stress conditions), *BnPAP1* was induced and formed MBW complexes to activate transcription of anthocyanin biosynthetic genes (e.g., *BnDFR*, *BnLDOX*, and *BnUF3GT*) and promote anthocyanin accumulation. BnCPC was up-regulated under stress conditions, and competed with BnPAP1 to interact with BnTT8/BnEGL3, thus affecting the MBW complex formation. BnCPC also inhibited the MBW complex-induced *BnCPC* activity, and formed a feedback loop with MBWs to regulate anthocyanin biosynthesis. Arrows and blunt arrows represent positive and negative regulation, respectively

In summary, we propose a working model of BnCPC inhibiting anthocyanin biosynthesis in *B. napus* (Fig. 8). Under non-inducible growth conditions (e.g., dark condition), the anthocyanin activator *BnPAP1* was expressed at a low level while *BnCPC* was continuously expressed to repress the formation of MBW complexes, thereby inhibited anthocyanin accumulation. Under anthocyanin inducible conditions (e.g., low temperature stress), BnPAP1 was induced and formed MBW complexes to activate transcription of anthocyanin biosynthetic genes (e.g., *BnDFR*, *BnLDOX*, and *BnUF3GT*) and promote anthocyanin accumulation. Meanwhile, BnCPC was up-regulated under stress conditions, and competed with BnPAP1 to interact with bHLHs (BnTT8 and BnEGL3), thus affected the MBW complex formation. Furthermore, BnCPC inhibited the MBW complex-induced *BnCPC* activity, and formed a feedback loop with MBWs to regulate anthocyanin biosynthesis.

## Conclusions

In this study, an R3-MYB repressor BnCPC was identified with functions in regulating anthocyanin accumulation in rapeseed. BnCPC could repress anthocyanin biosynthesis by competing with BnPAP1 to interact with bHLHs (BnTT8 and BnEGL3) and affect the formation of MBW complexes, and repress the expression of anthocyanin biosynthetic genes (e.g., *BnDFR*) that activated by MBW complexes. Meanwhile, the MBW complexes could induce the expression of *BnCPC*. In general, this research revealed a negative feedback loop of BnCPC and MBW complexes that controlled anthocyanin biosynthesis in *B. napus*, which will be helpful to understand the regulatory mechanisms of R3-MYB repressors on anthocyanin accumulation.

## Materials and methods

### Plant materials and growth conditions

The *B. napus* line J9712 was used as transgenic acceptor and control. Both J9712 and transgenic lines were grown in the experimental field in Yangzhou University (Jiangsu, China) for reproduction. For gene expression pattern analysis under cold, light, and dark conditions, the 4-day-old seedlings of J9712 grown in 1/2 MS medium in dark condition, were transferred to anthocyanin inducible (continuous light and low temperature at 10 °C, or continuous light and normal temperature at 23 °C) and non-inducible (continuous dark and normal temperature at 23 °C) growth conditions, respectively [73, 74]. Then, three replicates of ten seedlings were collected at 0 h, 1 h, 3 h, 6 h, 9 h, 12 h, 24 h, and 48 h after treatment, and stored at − 80 °C for use. Furthermore, both J9712 and transgenic lines were grown under different stress conditions for seven days to analyze the *BnCPC* function in regulating anthocyanin biosynthesis. For cold stress, seeds were germinated in 1/2 MS medium and grown at 10 °C, 16 h light/8 h dark, using the seedlings grown at 23 °C as control. For other stresses, seedlings were grown in 1/2 MS containing 10% sucrose or 30 μM JA, or solid Hoagland medium with 3 mM or 0.3 mM NH_4_NO_3_ under 23 °C, 16 h light/8 h dark cycles. The seedlings were pooled as mentioned above, and stored at − 80 °C for gene expression analysis and anthocyanin content measurement. *Nicotiana benthamiana* were grown in a light incubator (22 °C, 16 h light/8 h dark), and the 4-week-old plants were used for transient expression assays.

### Sequence and phylogenetic analysis

The protein sequences of *BnCPC* were downloaded from rapeseed genome database (http://www.genoscope.cns.fr/brassicanapus/), and the protein sequences of other known MYB repressors were downloaded from NCBI (https://www.ncbi.nlm.nih.gov/). Sequence conservation and phylogenetic analysis were conducted as reported before [75]. Multiple sequence alignment was performed with ClustalX (http://www.clustal.org/clustal2/). The phylogenetic tree was constructed with MEGA 7.0 (https://www.megasoftware.net/), using the neighbor-joining method.

### Subcellular localization

To determine the subcellular localization of BnCPC, the full-length coding sequence (CDS) of *BnCPC* (BnaC04g50810D) was cloned from *B. napus* cv. ‘Darmor-*bzh*’ and ligated into pEGAD vector to construct *35S::eGFP-BnCPC*. The *35S::eGFP-BnCPC* and *35S::eGFP* were transferred into *Agrobacterium tumefaciens* strain GV3101 by electroporation, and injected into the abaxial epidermis of *N. benthamiana* leaves for transient expression in dark for 48 h. The florescence images were captured using a confocal laser-scanning microscope (TCS SP8 STED, Leica, Germany). All primers are listed in Additional file 2: Table S2.

### Stable transformation of *BnCPC* in *B. napus*

The CDS of *BnCPC* (BnaC04g50810D) was cloned into the pMDC83 vector to construct an overexpression vector of *BnCPC*. The *35S::BnCPC* construct was used for hypocotyl transformation in *B. napus* line J9712 via *A. tumefaciens* strain GV3101 [76]. The transgenic lines were selected with 50 μg/mL hygromycin and confirmed with PCR amplification. All primers are listed in Additional file 2: Table S2.

### Transient expression assay in tobacco leaves

For transient assay of transcription factors (e.g., BnPAP1, BnTT8, BnEGL3, BnTTG1, and BnCPC) in regulating anthocyanin biosynthesis, the CDSs were cloned into p35S vectors and introduced into *A. tumefaciens* GV3101 strain. The primers are listed in Additional file 2: Table S2. The different combinations of constructs were co-transformed into tobacco leaves. After overnight incubation in the dark and grown under normal photoperiod for eight days, the leaf samples around the injection site were sampled for anthocyanin content and qRT-PCR analysis [70].

### Anthocyanin measurement

The anthocyanin content was measured according to the previous report with some modifications [77]. Briefly, 50 mg leaf sample was powdered and incubated in 200 μL extraction buffer (methanol containing 1% HCl) overnight at 4˚C in the dark. After centrifugation at 13,000 rpm for 10 min, the absorbance of the supernatant was measured at 530 and 657 nm. The relative anthocyanin content was calculated by (A_530_-0.25 × A_657_)/gram fresh weight. Three biological replicates were included for anthocyanin measurement.

### Y2H assay

The CDSs of *BnTT8*, *BnEGL3*, and *BnCPC* were cloned into pGADT7 (AD) and pGBKT7 (BD). The primers are listed in Additional file 2: Table S2. The AD and BD vectors harboring target genes were co-transformed into yeast strain AH109 according to the manufacturer’s instructions of the Matchmaker Gold Yeast Two-Hybrid system (Clontech, Japan). The protein interactions were detected on SD-Trp/-Leu/-His/-Ade + X-α-Gal medium, using pGBKT7-53 and pGADT7-T as positive control, pGBKT7-Lam and pGADT7-T as negative control [78].

### BiFC assay

The CDSs of *BnCPC*, *BnTT8*, and *BnEGL3* were cloned into pVYNE and pVYCE to fuse with the N- or C-terminal fragments of YFP. The primers are listed in Additional file 2: Table S2. All constructs were transformed into *A. tumefaciens* strain GV3101, and different combinations of BiFC constructs were co-transformed into 4-week-old *N. benthamiana*. For competitive binding assay, *35S*::*BnCPC* was co-transformed with different combinations of BiFC constructs. After culturing in dark for 48 h, the florescence images were observed using a confocal fluorescence microscope (TCS SP8 STED, Leica, Germany).

### GST pull-down assay

The CDSs of *BnCPC* and *BnPAP1* were cloned into the GST-tag-containing vector pGEX6p-1, while *BnTT8* and *BnEGL3* were cloned into His-tag-containing vector pET30a. Furthermore, *BnCPC* was also cloned into pET30a for competitive binding assay. All primers used are listed in Additional file 2: Table S2. The GST-BnCPC, GST-BnPAP1, His-BnCPC, His-BnTT8, and His-BnEGL3 constructs were transformed into *Escherichia coli* strain BL21 (DE3). The GST, GST-BnCPC, His-TT8 or His-EGL3 were immobilized using the ProteinIso^®^ GST Resin or ProteinIso^®^ Ni-NTA Resin (TransGen Biotech, Beijing, China). For pull-down assay, immobilized GST or GST-BnCPC were incubated with His-BnTT8 or His-BnEGL3 proteins at 4 °C for 2 h. For competitive binding assays, 5 μg of GST-PAP1 mixed with 5, 15, or 25 μg His-BnCPC were incubated with 5 μg of immobilized His-TT8 or His-EGL3 for 2 h. Beads were washed three times with the pull-down buffer. Proteins retained on beads were eluted by SDS-PAGE loading buffer and boiled for 5 min, then detected by anti-GST or anti-His.

### Dual-luciferase reporter assay

The dual-luciferase reporter assay was conducted in tobacco leaves as described in previous reports [79]. The *BnCPC* and *BnDFR* promoters were cloned into the pGreenII 0800-LUC vector to generate *proBnCPC:LUC* and *proBnDFR:LUC* as the reporters, respectively. The CDSs of *BnCPC*, *BnTT8*, *BnEGL3*, *BnPAP1*, and *BnTTG1* were inserted into pGreenII 62-SK as the effectors. All constructs were transformed into *A. tumefaciens* strain GV3101 containing a helper plasmid pSoup. The tobacco plants were grown in a growth chamber with 16 h of daylight. The reporters and effecters were mixed in ratios of 2:8, 2:4:4, or 2:3:3:3, and the different combinations of *A. tumefaciens* were injected into tobacco leaves. Two days after cultured under 16 h light/8 h dark and 22 °C, the injected leaves were collected for firefly luciferase (LUC) and renilla luciferase (REN) activity analysis, using the Dual Luciferase Reporter Assay Kit (Vazyme, Nanjing, China) and a Tecan Infinite M200 Pro luminometer (TECAN, Männedorf, Switzerland). Six independent biological replicates were included for dual-luciferase reporter assay. All primers are listed in Additional file 2: Table S2.

### Gene expression analysis

Three replicates of seedling samples of two *BnCPC* overexpression lines (OE-CPC-6, OE-CPC-11) and J9712 grown at 10 °C and 23 °C were collected for RNA-seq analysis [77]. Total RNA isolation and cDNA synthesis were performed with RNA isolator Total RNA Extraction Reagent (Vazyme, Nanjing, China) and the HiScript III RT SuperMix (Vazyme, Nanjing, China), respectively. PowerUp SYBR Green Master Mixes (Thermo, Waltham, MA, USA) and a StepOnePlus Real-Time PCR System (Thermo, Waltham, MA, USA) were used for qPCR analysis. The relative gene expression was calculated with the 2^−△△Ct^ method [80], using *B. napus Actin-7* (NC_027775.2) or *N. benthamiana Actin* (JQ256516.1) as internal controls. The qPCR primers are listed in Additional file 2: Table S2. The relative expression level of different *BnCPC* homologues in different tissues of rapeseed was obtained from transcriptome database BnTIR (http://yanglab.hzau.edu.cn/BnTIR) [81].

### Statistical analysis

All the data were expressed as mean ± SD. Statistical analysis was performed using SPSS 19.0. Independent-samples *t*-test was used to analyze significant difference between two samples. One-way ANOVA was carried out to compare statistical differences among groups with Duncan’s test. Significant differences were marked at *p* < 0.05 and *p* < 0.01 level.

## Supplementary Information

**Additional file 1: Figure S1.** The temporospatial expression pattern (A) and subcellular localization (B) of BnCPC. GFP fluorescence was shown in green. Bars = 10 μm. DAF, days after flowering; GFP, green fluorescent protein. **Figure S2.** qPCR analysis of *BnCPC* expression in overexpression lines of rapeseed. **Figure S3.** DEGs related to flavonoid biosynthetic processes. **Figure S4.** BnCPC repressed anthocyanin accumulation under different anthocyanin-inducible conditions. (A) Phenotype of J9712 and *BnCPC* overexpression (OE-CPC) lines under sucrose, JA, low nitrogen (LN), and high nitrogen (HN) treatments. (B) Anthocyanin content in extracts from seedlings in (A). (A_530_-0.25 × A_657_)/gram fresh weight was considered as the relative anthocyanin content. Three biological replicates were performed, and 10 plants were pooled as one replicate. FW, fresh weight. Values represented the mean ± SD (*n* = 3). Different letters represented statistically significant differences (one-way ANOVA, *p* < 0.05). (C) The expression level of *BnDFR*, *BnLDOX*, and *BnUF3GT* in seedlings from (A). Expression levels were standardized to *B. napus actin-7* (NC_027775.2), and the expression levels of J9712 under CK or HN were set at 1. Values represented the mean ± SD (*n* = 3).

**Additional file 2: Table S1.** DEGs related to flavonoid biosynthetic processes. **Table S2.** Primers used in the present study.

## Data Availability

All the data pertaining to the present study have been included in the tables and figures of the manuscript, and the authors are pleased to share all the data and plant materials.

## Abbreviations

MBW: MYB-bHLH-WD40
EBGs: Early biosynthesis genes
LBGs: Late biosynthesis genes
*CHS*: *Chalcone synthase*
*CHI*: *Chalcone isomerase*
*F3H*: *Flavanone 3-hydroxylase*
*F3′H*: *Flavanone 3′-hydroxylase*
*DFR*: *Dihydroflavonol 4-reductase*
*LDOX*: *Leucoanthocyanidin dioxygenase*
*UF3GT*: *UDP-glucose: flavonoid 3-O-glucosyltransferase*
PAs: Proanthocyanidins
EAR: Ethylene-responsive element-associated amphiphilic repression
DEGs: Differentially expressed genes
JA: Jasmonic acid
HN: High nitrogen
Y2H: Yeast two-hybrid
BiFC: Bimolecular fluorescence complementation
YFP: Yellow fluorescent protein
LN: Low nitrogen
CDS: Coding sequence
AD: pGADT7
BD: pGBKT7
LUC: Firefly luciferase
REN: Renilla luciferase
