# Supplementary figures and images for: An R3-MYB repressor, BnCPC forms a feedback regulation with MBW complex to modulate anthocyanin biosynthesis in Brassica napus
Source: Biotechnol Biofuels Bioprod. 2022 Nov 29;15:133. doi: 10.1186/s13068-022-02227-6 (PMC9706894; doi:10.1186/s13068-022-02227-6)

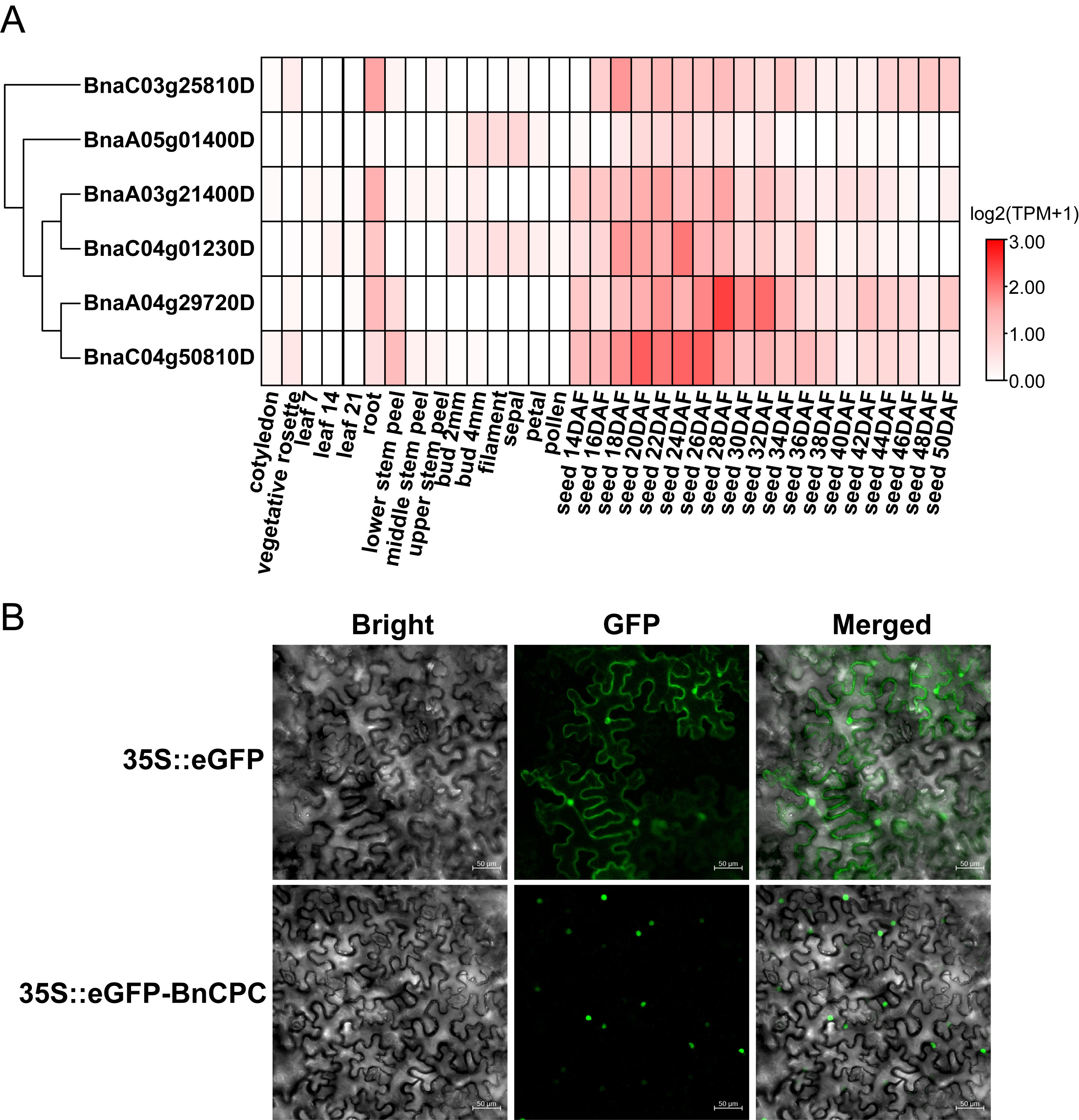

Supplement: Supplementary file 1 — Additional file 1: Figure S1. The temporospatial expression pattern (A) and subcellular localization (B) of BnCPC. GFP fluorescence was shown in green. Bars = 10 μm. DAF, days after flowering; GFP, green fluorescent protein. Figure S2. qPCR analysis of BnCPC expression in overexpression lines of rapeseed. Figure S3. DEGs related to flavonoid biosynthetic processes. Figure S4. BnCPC repressed anthocyanin accumulation under different anthocyanin-inducible conditions. (A) Phenotype of J9712 and BnCPC overexpression (OE-CPC) lines under sucrose, JA, low nitrogen (LN), and high nitrogen (HN) treatments. (B) Anthocyanin content in extracts from seedlings in (A). (A530-0.25 × A657)/gram fresh weight was considered as the relative anthocyanin content. Three biological replicates were performed, and 10 plants were pooled as one replicate. FW, fresh weight. Values represented the mean ± SD (n = 3). Different letters represented statistically significant differences (one-way ANOVA, p < 0.05). (C) The expression level of BnDFR, BnLDOX, and BnUF3GT in seedlings from (A). Expression levels were standardized to B. napus actin-7 (NC_027775.2), and the expression levels of J9712 under CK or HN were set at 1. Values represented the mean ± SD (n = 3). [file 13068_2022_2227_MOESM1_ESM.zip › Additional file 1/13068_2022_2227_MOESM1_ESM.jpg]

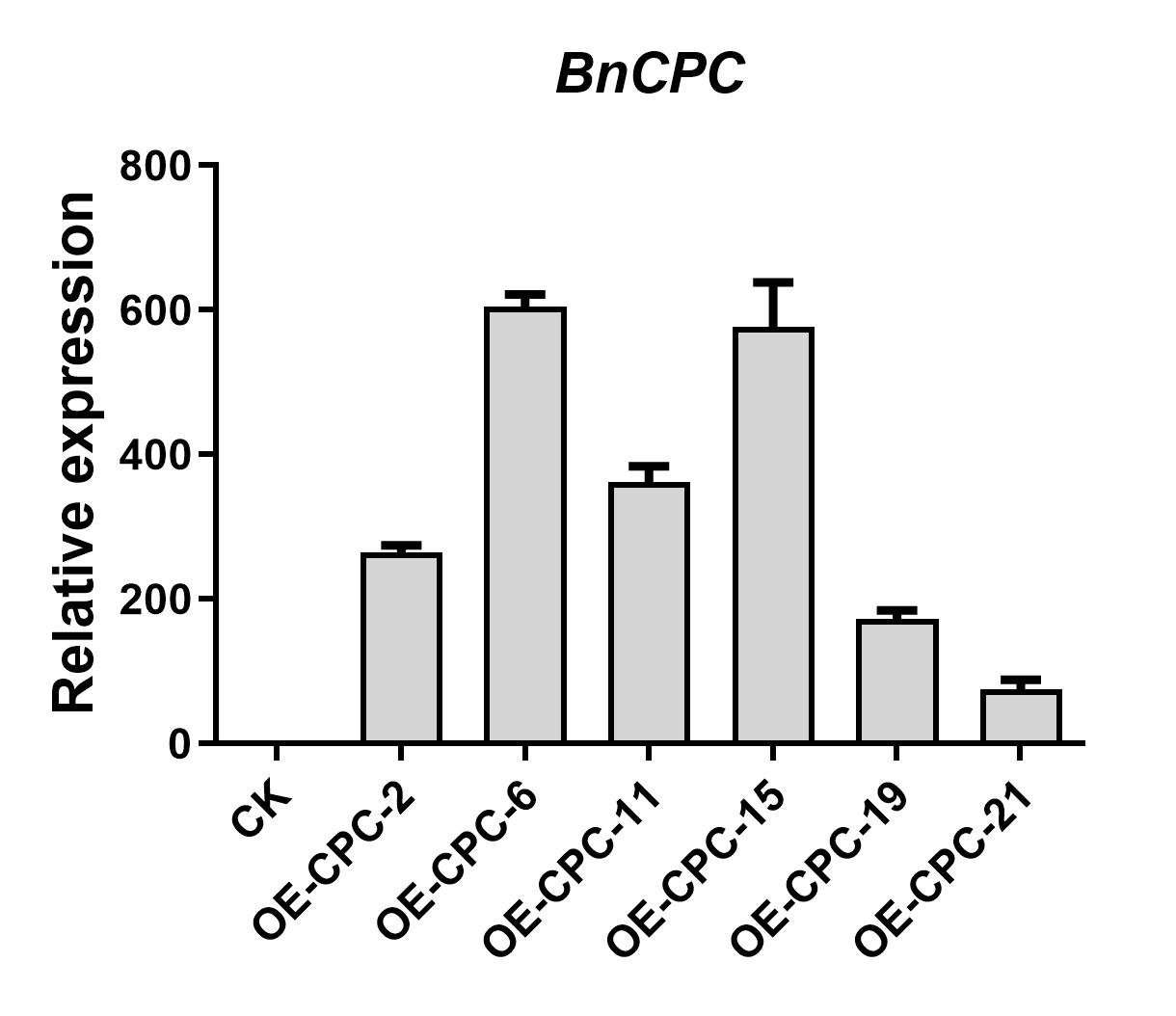

Supplement: Supplementary file 1 — Additional file 1: Figure S1. The temporospatial expression pattern (A) and subcellular localization (B) of BnCPC. GFP fluorescence was shown in green. Bars = 10 μm. DAF, days after flowering; GFP, green fluorescent protein. Figure S2. qPCR analysis of BnCPC expression in overexpression lines of rapeseed. Figure S3. DEGs related to flavonoid biosynthetic processes. Figure S4. BnCPC repressed anthocyanin accumulation under different anthocyanin-inducible conditions. (A) Phenotype of J9712 and BnCPC overexpression (OE-CPC) lines under sucrose, JA, low nitrogen (LN), and high nitrogen (HN) treatments. (B) Anthocyanin content in extracts from seedlings in (A). (A530-0.25 × A657)/gram fresh weight was considered as the relative anthocyanin content. Three biological replicates were performed, and 10 plants were pooled as one replicate. FW, fresh weight. Values represented the mean ± SD (n = 3). Different letters represented statistically significant differences (one-way ANOVA, p < 0.05). (C) The expression level of BnDFR, BnLDOX, and BnUF3GT in seedlings from (A). Expression levels were standardized to B. napus actin-7 (NC_027775.2), and the expression levels of J9712 under CK or HN were set at 1. Values represented the mean ± SD (n = 3). [file 13068_2022_2227_MOESM1_ESM.zip › Additional file 1/13068_2022_2227_MOESM2_ESM.jpg]

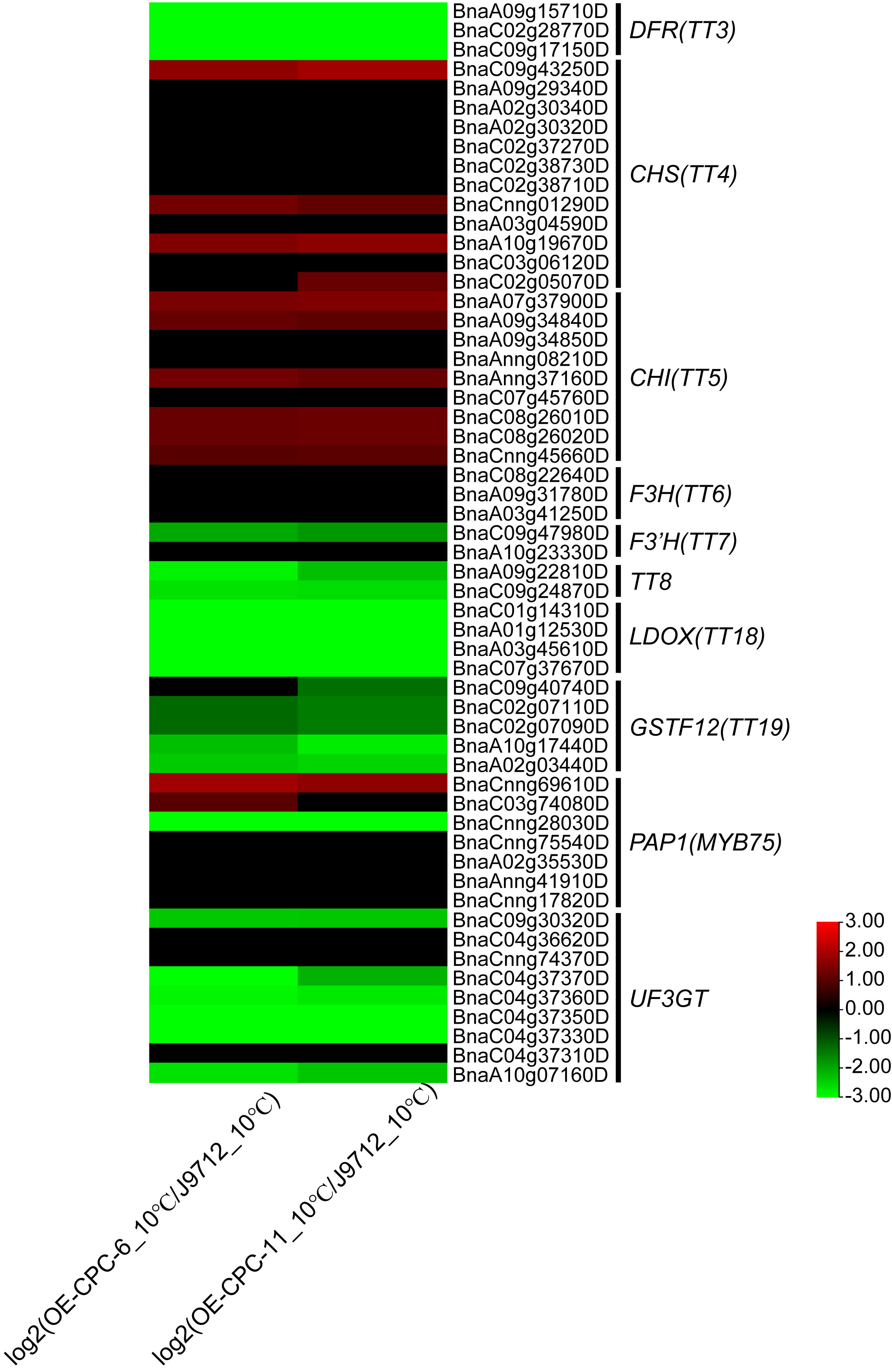

Supplement: Supplementary file 1 — Additional file 1: Figure S1. The temporospatial expression pattern (A) and subcellular localization (B) of BnCPC. GFP fluorescence was shown in green. Bars = 10 μm. DAF, days after flowering; GFP, green fluorescent protein. Figure S2. qPCR analysis of BnCPC expression in overexpression lines of rapeseed. Figure S3. DEGs related to flavonoid biosynthetic processes. Figure S4. BnCPC repressed anthocyanin accumulation under different anthocyanin-inducible conditions. (A) Phenotype of J9712 and BnCPC overexpression (OE-CPC) lines under sucrose, JA, low nitrogen (LN), and high nitrogen (HN) treatments. (B) Anthocyanin content in extracts from seedlings in (A). (A530-0.25 × A657)/gram fresh weight was considered as the relative anthocyanin content. Three biological replicates were performed, and 10 plants were pooled as one replicate. FW, fresh weight. Values represented the mean ± SD (n = 3). Different letters represented statistically significant differences (one-way ANOVA, p < 0.05). (C) The expression level of BnDFR, BnLDOX, and BnUF3GT in seedlings from (A). Expression levels were standardized to B. napus actin-7 (NC_027775.2), and the expression levels of J9712 under CK or HN were set at 1. Values represented the mean ± SD (n = 3). [file 13068_2022_2227_MOESM1_ESM.zip › Additional file 1/13068_2022_2227_MOESM3_ESM.jpg]

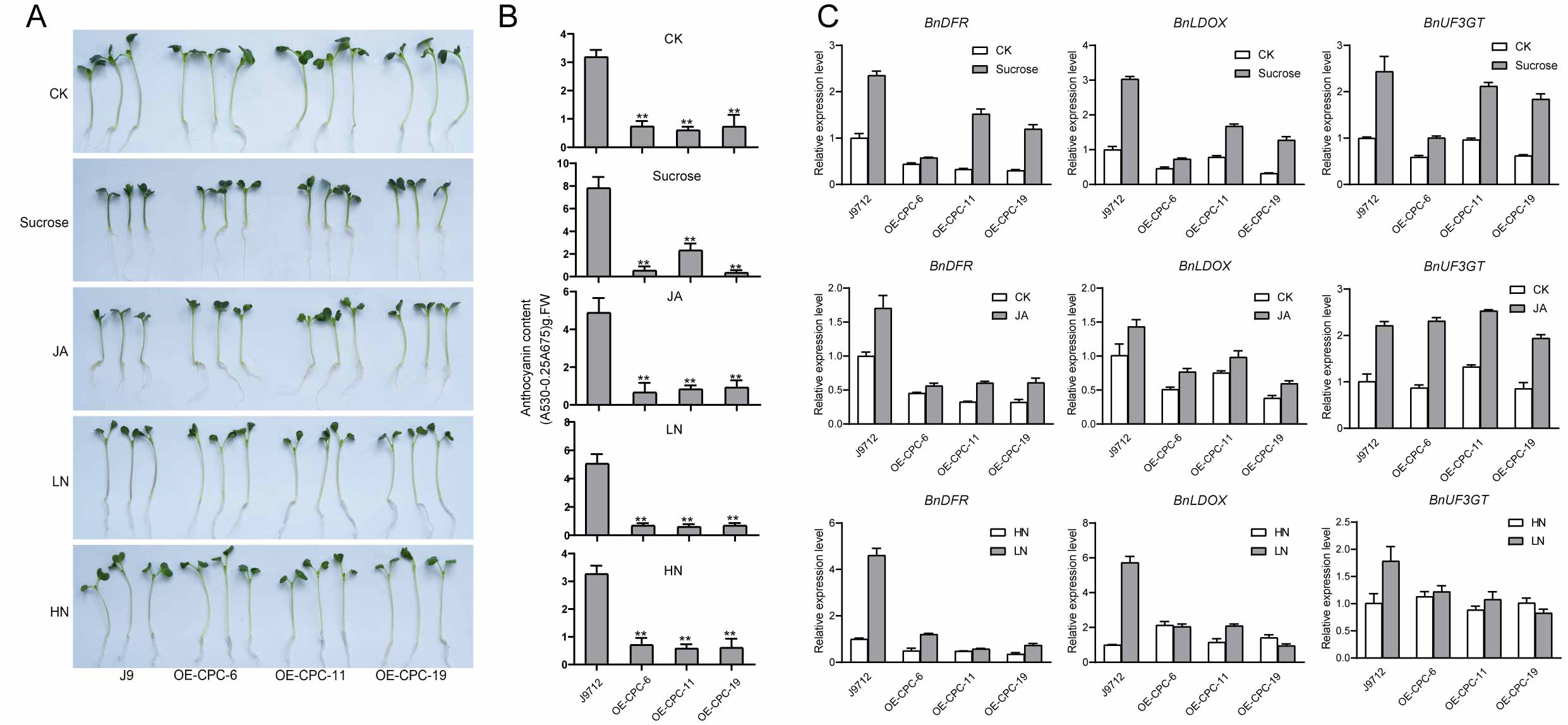

Supplement: Supplementary file 1 — Additional file 1: Figure S1. The temporospatial expression pattern (A) and subcellular localization (B) of BnCPC. GFP fluorescence was shown in green. Bars = 10 μm. DAF, days after flowering; GFP, green fluorescent protein. Figure S2. qPCR analysis of BnCPC expression in overexpression lines of rapeseed. Figure S3. DEGs related to flavonoid biosynthetic processes. Figure S4. BnCPC repressed anthocyanin accumulation under different anthocyanin-inducible conditions. (A) Phenotype of J9712 and BnCPC overexpression (OE-CPC) lines under sucrose, JA, low nitrogen (LN), and high nitrogen (HN) treatments. (B) Anthocyanin content in extracts from seedlings in (A). (A530-0.25 × A657)/gram fresh weight was considered as the relative anthocyanin content. Three biological replicates were performed, and 10 plants were pooled as one replicate. FW, fresh weight. Values represented the mean ± SD (n = 3). Different letters represented statistically significant differences (one-way ANOVA, p < 0.05). (C) The expression level of BnDFR, BnLDOX, and BnUF3GT in seedlings from (A). Expression levels were standardized to B. napus actin-7 (NC_027775.2), and the expression levels of J9712 under CK or HN were set at 1. Values represented the mean ± SD (n = 3). [file 13068_2022_2227_MOESM1_ESM.zip › Additional file 1/13068_2022_2227_MOESM4_ESM.jpg]
